# Supplementary material for: Caffeine, alcohol, khat, and tobacco use during pregnancy in Butajira, South Central Ethiopia
Source: PLoS One. 2020 May 8;15(5):e0232712. doi: 10.1371/journal.pone.0232712 (PMC7209255; doi:10.1371/journal.pone.0232712)
Supplement: Attachment — Submitted filename: Response to Reviewers.docx [file pone.0232712.s002.docx]

PONE-D-19-29554
Caffeine, Alcohol, Khat, and Tobacco Use during Pregnancy in Butajira, South Central Ethiopia
PLOS ONE

**Responses to the Academic Editor**

Dear Academic Editor,

Thank you for your valuable questions and suggestions. We have tried to respond the queries you raised on the method and limitation sections line by line as follow:

1. Please ensure that your manuscript meets PLOS ONE's style requirements, including those for file naming. The PLOS ONE style templates can be found at

<http://www.plosone.org/attachments/PLOSOne_formatting_sample_main_body.pdf> and <http://www.plosone.org/attachments/PLOSOne_formatting_sample_title_authors_affiliations.pdf>

**Response:**

- Thank you! We have prepared our manuscript as per the PLOS ONE’s guideline.

2. Please address the following:

- Please ensure you have thoroughly discussed any potential limitations of this study within the Discussion section. For example, the lack of follow-up with these participants does not allow for correlations to be made between substance use and birth weight.

**Response:**

- Dear academic editor Thank you! We incorporated it as “There was no follow up of pregnant women. Due to this, the study could not assess exist of correlation between substance use and birth weight” (Check on discussion section, page 29, lines 468-470).

- Please include additional information regarding the survey or questionnaire used in the study and ensure that you have provided sufficient details that others could replicate the analyses. For instance, if you developed a questionnaire as part of this study and it is not under a copyright more restrictive than CC-BY, please include a copy, in both the original language and English, as Supporting Information. In addition, please provide further details on the pre-testing of this questionnaire - i.e. how many participants were involved and from where were they recruited.

**Response:**

- Regarding the issues raised on the questionnaire, we did not develop a questionnaire. The questionnaire was adapted from Caffeine Consumption Questionnaire-Revised (CCQ-R), Alcohol Use Disorder Identification Test (AUDIT), and Global Adult Tobacco Survey (GATS), EDHS 2016 for caffeine, alcohol consumption, tobacco use and khat chewing assessment, respectively (Check on materials and methods section, page 8, lines 152-156).
- Concerning the pre-test, it was done on 21 pregnant women who recruited from out of the actual study area (Check on materials and methods section, page 11, lines 213-218).

- Please provide additional details regarding participant consent. In the ethics statement in the Methods and online submission information, please ensure that you have specified how verbal consent was documented and witnessed.

**Response:**

- Regarding the verbal consent, the verbal consent was documented by the interviewer on the prepared consent form attached with the questionnaire (Check on materials and methods section, page 12, lines 245 & 246).

- Please provide further details of your pilot study. This is mentioned in the text with no further information or reference to a published work. Thank you for your attention to these queries.

**Response:**

- Dear editor thank you! We conducted a pilot study before the actual data collection period to obtain the portion of coffee in the drink of coffee with milk. We recruited 12 households for the pilot study. Based on the pilot study we conducted, the estimated proportion of coffee in the drink of coffee served with milk was 0.7365 (Check on materials and methods section, page 9, lines 178-181).

**Response to Reviewer #1**

Dear Reviewer,

Thank you for your valuable questions and suggestions. We have tried to address the queries you raised on the introduction, result, method and discussion sections as follow:

a. I think the study objective should be assess the prevalence of caffeine and alcohol consumption, khat chewing, and tobacco and predictors of caffeine consumption among pregnant women in South Central Ethiopia rather than "determine the magnitudes of caffeine and alcohol consumption, khat chewing, and tobacco use during pregnancy and identify factors associated with excess caffeine consumption".

**Response:**

- Thank you for your suggestion! It was corrected as “To assess the prevalence of caffeine and alcohol consumption, khat chewing, and tobacco use during pregnancy and identify factors associated with excess caffeine consumption” (Check on the abstract section, page 2, line 27-29).

b. The introduction is very long, some data are redundant. I suggest shortening and reviewing some paragraphs. Also, the last paragraph on page 5 would be better suited to the discussion.

**Response:**

- Thank you for your suggestion! To make the introduction more informative, it becomes somehow long as you said. If we shorten it, it will not be informative. That is why we obliged to narrate it as such. Nevertheless, we have tried to make it short.

c. The discussion should focus on the physiological and sociodemographic factors that may contribute to the increase in maternal caffeine intake. The study results should be confronted with others in the literature. However, the discussion has a strong focus on the adverse effects of maternal habits on the fetus and child that I consider to be more suitable for the introduction.

**Response:**

- Thank you for your insight! We have discussed those factors which are significantly associated with excess caffeine intake. In addition, we incorporated the effects of caffeine intake to show the implication of excess caffeine intake during pregnancy on the fetus (Check on discussion section, page 28, line 444-452).

d. Was the design effect used for sample calculation?

**Response:**

- Because we have used stratified sampling technique, design effect was not considered during sample size determination.

e. Was the questionnaire validated?

**Response:**

- Dear reviewer, we did not develop a questionnaire. The questionnaire was adapted from Caffeine Consumption Questionnaire-Revised (CCQ-R), Alcohol Use Disorder Identification Test (AUDIT), and Global Adult Tobacco Survey (GATS), EDHS 2016 for caffeine, alcohol consumption, tobacco use and khat chewing assessment, respectively (Check on materials and methods section, page 8, lines 152-156).

f. It was not possible to quantify the concentration and the brand of caffeine products in this study. The author did not consider the strength of coffee as commonly consumed (weak, medium, or strong coffee). These limitations need to be included in the discussion.

**Response:**

- Thank you! We incorporated it as a limitation (Check on discussion section, page 29, line 459-462).

g. Could the lack of information about some medications to underestimate the amount of caffeine ingested by pregnant women?

**Response:**

- Yes, it underestimates the amount of caffeine consumption among pregnant women. Some drugs such as Aspirin contain caffeine as an ingredient. But, pregnant women mainly not took those drugs during pregnancy.

h. The literature report that social factors such as the family history of alcoholism, occupation of the head of the family, and higher parity are positively associated with high caffeine consumption. The author should comment on these limitations in the discussion.

**Response**:

- Dear reviewer, thank you for your insight. We have tried to search factors associated with excess caffeine consumption during pregnancy. But we could not find any study conducted on factors associated with excess caffeine consumption during pregnancy.

i. Please define ANC follow up abbreviation (antenatal care).

**Response:**

- Thank you! ANC follow up corrected as “antenatal care follow up” (Check on Result section, page 23, Table 7, between line 261 & 262).

j. Page 6: The sentence "The study was conducted in Butajira Rural Health Program (BRHP) site, which is found in Meskan and Mareko districts of Gurage Zone, SNNP region, South Central Ethiopia" is confusing. This needs to be more clearly described. Is the author saying the study was conducted in areas (kebeles) covered by BRHP?

**Response:**

- Yes and it is corrected as “The study was conducted in Kebeles covered by Butajira Rural Health Program (BRHP) which is found in Meskan and Mareko districts of Gurage Zone, SNNP region, South Central Ethiopia.” (Check on materials and methods section, page 6, line 123-125).

k. Page 6: I think the sentences "Butajira Rural Health Program is a Health Demographic Surveillance System (HDSS) Site for Addis Ababa University" and "BRHP comprises one urban and nine rural kebeles (a kebele is the smallest administrative unit in Ethiopia)" are confusing. Does the Butajira Rural Health Program is a Health Demographic Surveillance System (HDSS) site? Does the BRHP cover selected kebeles of the Meskan and Mareko district? Please clarify it.

**Responses:**

- Yes, Butajira Rural Health Program is a Health Demographic Surveillance System (HDSS) site (Check on materials and method, page 6 & 7, line 125-127).
- Yes, BRHP covers the selected kebeles of Meskan and Mareko district and clarified as suggested (Check on materials and method section, page 6 & 7, line 125-127).

l. Page 7: "The source population of the study was all pregnant women living at BRHP". I’m not sure of what you mean "living at BRHP". Please, clarify it.

**Response:**

- Thank you! It is corrected as “The source population of the study was all pregnant women living at Butajira Rural Health Program” (Check on materials and method section, page 7, line 131 & 132).

m. Page 7: "The list of pregnant women was obtained from the BRHP database". How about the recruitment process? How they were initially contacted? Was there a visit to the participant’s home? How about the exclusion or inclusion criteria?

**Responses:**

How about the recruitment process?

- The total number of pregnant women during the data collection period was 466 as obtained from the Butajira Rural Health Program data base. Sampling frame was prepared for each stratum and the samples were assigned for each stratum proportional to the number of pregnant women. Then, study subjects were selected from each stratum by using simple random sampling (SRS) technique (Check on materials and method section, page 7, line 142-150).

How they were initially contacted?

- The sampling frame was obtained from the Butajira Rural Health Program site. They continuously updated the database. Once we select the samples, the interview conducted at the respondents’ house (Check on materials and method section, page 7, line 145&146).

Was there a visit to the participant’s home? How about the exclusion or inclusion criteria?

- There was a visit to the participant’s home during the data collection time.
- Pregnant women living in the study area were included in the study. None of the pregnant women was excluded (Check on materials and method, page 7, line 132 & 133).

n. Page 11: I think the sentence "After data collection, the data were entered using Epi-data version 3.1 software to avoid an introduction of error while data entry" is redundant. This information is described in the next paragraph.

**Response:**

- Thank you! The statement “After data collection, the data were entered using Epi-data version 3.1 software to avoid an introduction of error while data entry” is removed as suggested (Check on materials and methods section, page 11, line 121).

o. Page 14: "However, Pepsi cola, energy drinks, and chocolates were not sources of caffeine among pregnant women in the study area pregnancy" I’m not sure of what you mean "sources of caffeine among pregnant women in the study area pregnancy ". Please, clarify it.

**Response:**

- Thank you! It is corrected as “However, Pepsi cola, energy drinks and chocolates were not the sources of caffeine among pregnant women in the study area” (Check on result section, page 15, line 275 & 276).

p. Page 22: The sentence “The possible reason for this high prevalence of excess caffeine intake during the first trimester of pregnancy needs further investigation” should be discussed. The literature report that in the first-trimester, behavioral changes occur aiming at enhancing personal care, and symptoms such as nausea or aversion to the coffee smell or taste can be quite common during pregnancy, reducing coffee consumption.

**Response**:

- Thank you for your insight! We discussed as “This finding is not in line with the previous literature. According a study in 2004, 96% of subjects decreased or quit drinking coffee during first trimester pregnancy. The possible reason for this discrepancy and high prevalence of excess caffeine intake during first trimester of pregnancy needs further investigation” (Check on discussion section, page 25, line 381-383).

q. Page 25: The statement “The current study showed that the odds of excessive caffeine consumption is approximately… at the richest wealth status compared to the odds among pregnant women at the poorest wealth status” seems no make much sense. Positive associations with poor women were seen. Besides, no significant association between middle income and coffee consumption were observed.
   **Response**:

- Thank you for your point of view! Though, there was a positive associations between excess caffeine consumption among poor women and excess caffeine consumption and no significant association between middle income and excess caffeine consumption were seen, the odds of excessive caffeine consumption is approximately four times higher among pregnant women at the richest wealth status as compared to the odds among pregnant women at the poorest wealth status. That is why we narrate and discussed it as such (Check on result section, page 22, line 342-347).

r. Page 25: “Due to this woman with richest wealth status might consume excess caffeine compared to woman with the poorest wealth status” and “As women wealth status increases, the chance of buying coffee and consuming caffeinated beverages might increase.” Poor people of rural areas do not consume coffee? Is coffee expensive in the studied areas? Does the studied area plant coffee?

**Responses:**

- We are not saying that poor people of rural areas do not consume coffee.
- Yes, coffee is expensive in the study area. It is around 3 dollar per kg.
- The studied area does not plant coffee.

s. Page 26: I’m not sure I understand this statement “Second, the study was conducted during the non-fasting season, and our report might overestimate the prevalence of excess caffeine consumption.” Is the author saying that women consume more caffeine because, in the fasting season, they can't do it? Please, clarify this statement.

**Responses**:

- They can drink it during the fasting season. But, the authors want to say that the frequency of drinking coffee per day becomes decrease during the fasting season and increase during the non-fasting season. Due to this the intake of excess caffeine consumption may underestimated during the fasting period. While it may be overestimated during the non-fasting period (Check on discussion section, page 29, line 463-465).

**Response to Reviewer #2**

Dear Reviewer,

Thank you for your valuable suggestions. We have tried to address the queries you raised on the method section as follow:

- The manuscript’s issue is the excessive use of caffeine and the consumption of alcohol, cigarette, and khat during pregnancy, and their adverse effects on the fetus's health. The theme is relevant and actual once it is a public health problem in many countries in the world. It is more critical in developing countries, in which many other socioeconomic risk factors can impact the pregnancy so, it is important to produce scientific information specifically from these countries. The authors present global statistical data that strengthen the importance of the issue.

**Response:**

- Thank you!
- The abstract summarizes the arguments in an adequate way.
  Method: the study area, study population, and sample size determination and sampling procedure are well described. A pilot study and a pretest were conducted and, a “two days training” was done to the data collectors and supervisors before the accomplishment of the study.

**Response:**

- Thank you!
- The authors considered that “the accessibility of substances might vary based on residence and agro-ecological zones”. It could be interesting to add information about how this accessibility could vary and which are the characteristics of these zones and their impact on drug use that could justify this division. Additionally, there are no comments about this point in the Results or Discussion sessions.

**Response:**

- Dear reviewer thank you for your comment! The accessibility of substances at rural and urban areas may not be the same. For instance: Tobacco may not be accessible at rural areas as such to the urban areas. In addition, Khat grows mainly in highland than lowlands. Due to these reasons, the accessibility of substances use may vary based on residence and agro ecological zones (Check on materials and method, page 7, line 142 & 143).
- The results are well described, and the Discussion encompasses the main findings. The Conclusion is pertinent.

**Response:**

- Thank you!

**Response to Reviewer #3**

**Reviewer #3 comments**

In the submitted manuscript the authors investigated the prevalence of caffeine, alcohol, khat, and tobacco use among 352 pregnant women in Ethiopia. They also investigated the main factors associated with the excess of caffeine intake among those women. The consumption of these substances during pregnancy may pose a risk to the health of mothers and newborns. Since information on the use of these substances during pregnancy in developing countries like Ethiopia is very scarce, this manuscript can bring new findings to the subject. However, to be published this manuscript needs some improvements.

Dear Reviewer,

Thank you for your valuable questions and suggestions. We have tried to address the major and minor comments you raised as follow:

**Response to Major Comments:**

- The authors could have moved forward and, in addition to assess the use of caffeine, alcohol, khat, and tobacco during pregnancy, they could also have investigated the association between the use of these substances and adverse birth outcomes in the studied population. Although this association has been extensively studied, very few is known about this relationship under the socioeconomic conditions expected to be found in developing countries like Ethiopia.

**Response:**

- Thank you! We appreciate your point of view. In fact we did not assess the data related with adverse pregnancy outcomes. Due to this we cannot investigate any association between the use of these substances and adverse birth outcomes in the studied population. We mentioned this as a limitation in the discussion section (Check on discussion section, page 29, line 468-470).
- English needs major revision.

**Response:**

- Thank you! We have revised the entire document.

**Response to Minor comments**

- Page 3, line 54: The definition of "substance use" was retrieved from a website. I recommend that the authors search for a definition on a paper or book. In addition, I believe that authors meant to define substance abuse, not use.

**Response:**

- Thank you for your recommendation. It would be nice if the source is from paper or book. But, we could not found any definition from book or paper. It is a medical dictionary definition, thus we decided to use as it is.
- The authors mean to define substance use. Because, during pregnancy even substances use has an effect on the developing fetus.
- Page 3, line 63: This submitted version still shows (ref), which I assumed as a missing reference.

**Response:**

- Thank you! We incorporated the missed reference (Check on introduction section, page 4 line 64).
- The authors obtained the amount of caffeine intake from several sources through a two-24-hr recall survey, one in a weekday and another on a weekend day. Then, they calculated an average intake by summing the two-24-hr, and dividing by 2. Did authors test for differences in the caffeine intake between weekdays and weekend days for the different sources?

**Response:**

- We did not test for differences in the caffeine intake between weekdays and weekend days for the different sources.
- The authors found that the factors significantly associated with excess consumption of caffeine were the richest wealth status and the first trimester of pregnancy. However, they failed to describe that, compared with the poorest; the poor wealth status was also associated with excess of caffeine intake. In addition, crude odds ratios also suggested that age and antenatal care follow up may also be associated with the outcome. Although such associations were not statistically significant in the adjusted analyses, they were borderline and deserve to be mentioned in the results’ section.

**Response:**

- Thank you! We described it as you suggested (Check on result section, page 22, lines 345-347).
