# Supplementary material for: Caffeine, alcohol, khat, and tobacco use during pregnancy in Butajira, South Central Ethiopia
Source: PLoS One. 2020 May 8;15(5):e0232712. doi: 10.1371/journal.pone.0232712 (PMC7209255; doi:10.1371/journal.pone.0232712)
Supplement: Attachment — Submitted filename: Response to Reviewers.docx [file pone.0232712.s003.docx]

**Response to the Academic Editor**

Dear Academic Editor,

We have tried to address the points raised on result presentation and interpretation during the review process line by line.

**Comment 1:**

- Please modify the materials and method section, page 7, line 131 & 132 to make it clear that the population of the study was "all pregnant women living in kebeles covered by BRHP".

**Response:**

- Thank you for your suggestion! We have modified it as “The source population of the study was all pregnant women living in *kebeles* covered by Butajira Rural Health Program” (Check on Material and Method section, page 7, line 139 & 140).

**Response to Reviewer #1**

Dear Reviewer,

Thank you for your invaluable concern. We have addressed the raised concerns on the result presentation and interpretation sections as follow:

1) Page 25: “The current study showed that the odd of excessive caffeine consumption is approximately… at the richest wealth status compared to the odds among pregnant women at the poorest wealth status”. According to manuscript Table 7, the adjusted OR for excessive caffeine consumption according to wealth status was:

Poor woman vs poorest one (OR: 3.63, 95%CI: 1.16, 11.32)

Rich woman vs poorest one (OR: 3.74, 95%CI: 1.17, 11.88)

Richest woman vs. poorest one (OR: 3.66, 95%CI: 1.13, 11.88)

Looking at the CIs, if the author hypothetically conducts the same study with a different sample, the true value (OR) of excessive intake of caffeine for poor women in relation to the poorest, may be between 1.16 and 11.32. For rich women compared to the poorest one, the true odds may be between 1.17 and 11.88, and for the richest women than the poorest woman, this true value might be between 1.13 and 11.88.

Regarding the issues raised above, is the Odds ratio from 3.63 different from 3.66 and different from 3.74? Besides, an OR of 2 means there is a 100% increase in the odds of an outcome with a given "exposure". In relation to the paper results, in terms of magnitude, is an increase of 263% different from 274% and different from 266%? Please explain why only the result for the richest woman group compared to the poorest ones were highlighted and discussed.

**Response:**

- Dear reviewer, we really thank you for your insight! We have admitted your concern. Thus, we have described the odds ratio of excessive caffeine consumption among women in the poor wealth quintile as compared to the women in the poorest wealth quintile (Check on result section, page 23, lines 360-369).
- In addition, we have revised the discussion section (Check on discussion section, page 29, lines 464-466 and lines 470 & 471).

2) Page 26: “...the authors want to say that the frequency of drinking coffee per day decreases during the fasting season and increases during the non-fasting season.”
During the fasting period, the types of food and drinks and their frequencies are changed (Masood et al. 2018). But I did not find any literature that states that religious fasting alters food patterns after fasting. Please, include references that support your statement.
Masood, S. N., Saeed, S., Lakho, N., Masood, Y., Ahmedani, M. Y., & Shera, A. S. (2018). Pre-Ramadan health seeking behavior, fasting trends, eating pattern and sleep cycle in pregnant women at a tertiary care institution of Pakistan. Pakistan journal of medical sciences, 34(6), 1326.

**Response:**

- Dear reviewer, sure, the authors want to say that the frequency of drinking coffee per day decreases during the fasting season and increases during the non-fasting season. Because, in Ethiopia, coffee is traditionally consumed under coffee ceremony and the ceremony is a minimum of three times a day. In each ceremony, a person will take three cups of coffee (Yoseph, 2013). Hence during fasting time, the number of ceremony decreases and this alter the daily coffee consumption. To the contrary, the frequency of coffee consumption increases during non-fasting season. Due to this reason our study might overestimate the prevalence of excessive caffeine consumption (Check on discussion section, page 30, lines 484-490).

**Response to Reviewer #3:**

**Comment:** I have carefully reviewed my comments and suggestions sent to the authors. I'm satisfied with the authors' answers.

- **Response:** Dear reviewer, we are thankful for your invaluable contribution in the improvement of this manuscript.
